# The association of dietary behaviors and practices with overweight and obesity parameters among Saudi university students

**DOI:** 10.1371/journal.pone.0238458

**Published:** 2020-09-10

**Authors:** Nabeel Kashan Syed, Mamoon Hussain Syed, Abdulkarim M. Meraya, Ahmed A. Albarraq, Mohamed Ahmed Al-kasim, Saad Alqahtani, Hafiz Antar Makeen, Ayesha Yasmeen, Otilia J. F. Banji, Mohamed Hassan Elnaem

**Affiliations:** 1 Pharmacy Practice Research Unit, Department of Clinical Pharmacy, Faculty of Pharmacy, Jazan University, Gizan, Kingdom of Saudi Arabia (K.S.A); 2 Department of Pharmacy Practice, Faculty of Pharmacy, International Islamic University, Malaysia, Kuantan, Pahang, Malaysia; San Raffaele Roma Open University, ITALY

## Abstract

**Background:**

Western dietary habits, coupled with a sedentary lifestyle, are potential contributors to the prevalence and rapid increase in the incidence of obesity in Saudi Arabia. This study aimed to investigate the association between students' weight status and their eating behaviors and practices. Another aim was to assess students' awareness of the health risks associated with obesity.

**Methods:**

A cross-sectional survey was conducted among a sample of 416 (53% male and 47% female) undergraduate students, aged 18–26 years old, between January 6 and April 6, 2019, from colleges of Health Sciences at Jazan University in the Kingdom of Saudi Arabia (K.S.A). Students completed a self‐administered questionnaire and recorded their measured anthropometric parameters.

**Results:**

The prevalence of overweight (20.4%) and obesity (14.9%) were relatively high among the participants. There were statistically significant associations between Body Mass Index (BMI) and the different settings of food consumption (i.e., dining on a table (or) in the Islamic way: squatting on the ground) (p<0.001)). BMI was also associated with students' dietary habits regarding consuming food, snacks, and drinking carbonated beverages while watching television (p<0.001), as well as consuming the same pattern of food/drink while watching television, playing video games on mobile phones or computers (p<0.001). Nearly most of the students were oblivious to the fact that metabolic syndrome, reproductive disorders, respiratory disorders along with liver and gallbladder diseases are some of the health risks associated with obesity.

**Conclusion:**

The prevalence of obesity and overweight were reasonably high in our study sample and were affected by several factors related to students' eating behaviors and practices. This warrants the need for rigorous and frequent health education interventions on healthy eating behaviors, dietary practices, with an emphasis on the importance of adopting an active, healthy lifestyle.

## Introduction

The World Health Organization (WHO) has identified obesity as a global health problem in which almost 1.5 billion adults were overweight in 2008, and an additional 200 million men and 300 million women were obese [1]. In 2016, it was estimated that more than 1.9 billion adults were overweight, while more than 650 million were thought to be obese [2].

The Kingdom of Saudi Arabia (K.S.A) has been experiencing unprecedented economic growth for the last couple of decades [3, 4]. This exponential economic growth has also resulted in massive changes in eating patterns, which has culminated in the food choices made becoming westernized with the passing of each day [3]. Moreover, these changes have led to considerable changes in the lifestyle as well as the living standards [4]. Consequently, this massive societal makeover has also influenced the diet, resulting in vast changes in dietary habits and social practices, leading to many unhealthy practices [4]. The increase in the rates of obesity are a consequence of drastic lifestyle changes, improvement in the socioeconomic status along with changing cultural trends [5]. Binge eating, eating fried food, and lack of physical activity have all been associated with a terrifying increase in obesity [6–10]. Food products high on preservatives are increasingly replacing traditional diet and diet containing fruits and vegetables that are rich in fiber [11, 12]. The change in the diet from the traditional healthy food to the more modern, westernized but unhealthy diet, is being attributed to a growing cause of obesity amongst the population of K.S.A [13–16].

College students are often busy with their studies and tend to spend a lot of time outside their homes and hence, are prone to fall victim to eating practices that are unhealthy, gradually leading to an increase in body weight [17]. WHO estimates the prevalence of obesity to be generally higher in women than in men [2]. Nonetheless, recent studies on college students reveal otherwise, showing increased rates of obesity in male students as compared to female students [17, 18].

This study aimed to investigate the association between students' weight status and their eating behaviors and practices. Another aim was to assess students' awareness of the health risks associated with obesity.

## Materials and methods

### Study design and study population

A cross-sectional survey was conducted among a sample of 416 (53% male and 47% female) undergraduate students, aged 18–26 years old, between January 6 and April 6, 2019, from colleges of Health Sciences at Jazan University in Saudi Arabia. The colleges included were Pharmacy, Medical, Dental, Nursing, Applied Health, and Public Health. The participants were selected by convenience sampling. Students completed a self‐administered questionnaire and recorded their measured anthropometric parameters.

### Inclusion and exclusion criteria

#### Inclusion criteria

Male and female students above the age of 18 and below 26, studying in colleges of Health sciences (Pharmacy, Medical, Dental, Nursing, Applied health and Public health), Jazan university. They were deemed eligible for inclusion if they had a clear understanding of either Arabic (or) English language, were willing to participate in the research, agree to provide informed consent, were willing to complete the questionnaire, and were willing to provide their anthropometric readings.

#### Exclusion criteria

Students were excluded from taking part in the study if they did not meet the inclusion criteria.

### Data collection tools

A validated 26-item questionnaire, along with anthropometric measurements, were used as tools for data collection.

#### Development, validation and translation of the questionnaire

The initial draft of the questionnaire was prepared by the authors after careful and prolonged deliberation. A five member independent expert committee consisting of two skilled, experienced physicians and three pharmacists was constituted to review the questionnaire for content and face validation. The questions that were deemed inappropriate by the expert committee were subsequently deleted. The final, approved version of the questionnaire consisted of 26 items, spread over 2 sections. Section—1 contained questions regarding the participants’ demographics and anthropometric measurements. Section—2 was further subdivided into questions related to the participants’ eating behaviors, eating habits, physical activity, and their awareness of the health risks associated with obesity.

We utilized a translation method called as the forward-backward word translation. The expert services of a bilingual professional translator having excellent and expert-level fluency in both Arabic and English languages was used to translate the 26-item questionnaire. He translated the English version of the questionnaire into Arabic. This Arabic translation was then reviewed by a bilingual study author, having great fluency in both Arabic and English languages. Any inconsistencies were subsequently discussed with the translator and resolved, and then a final version was prepared and approved. Lastly, the final approved Arabic version of the questionnaire was then back-translated into English by another bilingual author who until now, was kept unaware of the original English version of the questionnaire. Both the forward, as well as the backward translations, were then reviewed in detail among all the authors. The Arabic version of the questionnaire was given to a focus group of ten students to assess the ease of use and the completion time.

#### Data collection

The participants were recruited in the study by convenience sampling. Those who met the inclusion criteria were included in the study. We divided three of our authors into two data collection teams. One male author and two students were responsible for the participant recruitment and data collection of the male students and two female authors were responsible for doing the same for the female students. The recruitment and data collection was done by these teams during a three month period from January 6 and April 6, 2019. Permission and approval was sought from the concerned authorities prior to the college visits. Students were then reminded a day in advance on the social media groups regarding the upcoming visit. We approached the students in the morning before they had taken any breakfast. A mobile desk used to be set up, and the teams used to explain to the students the aim and the benefits of taking part in the research. Those willing to give their consent were given the option of selecting the language of the questionnaire they are most comfortable with (Arabic/English). After the completion of the questionnaire, their anthropometric measurements were taken. A weighing scale was used to record the weight (in kilograms) and a mobile stadiometer kept against a wall was used to measure the height (in centimeters) accurately. The readings were taken twice to increase accuracy and reduce intra observer variability. BMI was later calculated by entering the recoded values into an online NHLBI-NIH BMI calculator.

### Sample size

The sample size was determined as 351, on the basis of a 5% margin of error, 95% confidence interval, a population size of 4000 students and a 50% response distribution. We distributed the questionnaire to around 450 students, out of which 416 gave consent and completed the questionnaire as well as facilitated in the recording of their anthropometric measurements, giving a response rate of 92.44%.

### Ethical considerations

Appropriate ethics committee approval (Approval Number: 1861/253/1440) was taken from the Institutional Research Review & Ethics Committee (IRREC) of Jazan University, prior to the start of the study.

### Statistical analysis

Data were analyzed using Statistical Package for the Social Science (SPSS Inc., Chicago, IL., USA) statistical software (version 23). The result of the demographics section was expressed in terms of frequency, total percentage, means ± standard deviations. Cross tabulations with Pearson’s Chi-square were used to look for a statistically significant association between the variables. The alpha level was set at 0.05 to determine statistical significance. Multivariate logistic regression model was also used to examine any association between eating behaviors, eating practices with obesity as an outcome.

## Results

### Sample characteristics

The first section of the questionnaire included questions on the demographics of the study participants, the results of which are shown in Table 1. In the present study, a total of 416 Saudi students participated, out of which more than half [n = 219 (52.6%)] were male students and the rest [n = 197 (47.4%)] were female students. The average age of the respondents was found to be 21.01 ± 2.35 years. The highest percentage of the study participants were from the college of Pharmacy [n = 150 (36.1%)], while the lowest were from college of Public health [n = 38 (9.1%)]. With regards to the study year, fourth year [n = 122 (29.3%)] was the highest contributor, while the sixth year [n = 88 (21.2%)] contributed the least. More than half [n = 239 (57.5%)] of all the study participants resided in rural areas, while the remaining [n = 177 (42.5%)] resided in urban areas. Majority of the participants [n = 323 (77.6%)] were non–smokers, while current smokers [n = 76 (18.3%)] were less and only a few were past smokers [n = 17 (4.1%)]. The highest percentage [n = 216 (51.9%)] of the study participants belonged to the age group of (18–20) years, while the least percentage [n = 98(23.6%)] was from the age group of (24–26) years. The prevalence of overweight (20.4%) and obesity (14.9%) were relatively high among the study participants. Just over half of the participants (51.4%) were found to be in the normal weight group, while (13.2%) were underweight.

**Table 1:** Demographics / sample characteristics.

Variable	Options		Frequency		Percentage	
**Gender**	Male		219		52.6	
	Female		197		47.4	
**Age Group (in years)**	18–20		216		51.9	
	21–23		102		24.5	
	24–26		98		23.6	
**Nationality**	Saudi		416		100.0	
**College**	Pharmacy College		150		36.1	
	Medical College		70		16.8	
	Dental College		40		9.6	
	Nursing College		50		12.0	
	Applied Health College		68		16.3	
	Public Health		38		9.1	
**Level**	3rd Year		112		26.9	
	4th Year		122		29.3	
	5th Year		94		22.6	
	6th Year		88		21.2	
**Location**	Rural		239		57.5	
	Urban		177		42.5	
**Smoking History**	Non-Smoker		323		77.6	
	Current Smoker		76		18.3	
	Past Smoker		17		4.1	
**BMI**	Underweight		55		13.2	
	Normal		214		51.4	
	Overweight		85		20.4	
	Obese		62		14.9	
	**Minimum**	**Maximum**		**Mean**		**Std. Deviation (±)**
**Age–Males**	18	25		21.04		± 2.35
**Age–Females**	18	25		20.97		± 2.34
**Height (cms)–Males**	160.9	188.7		175.03		± 5.43
**Height (cms)–Females**	141.2	174.2		158.62		± 7.91
**Weight (kgs)–Males**	43	135		77.68		± 16.94
**Weight (kgs)–Females**	36.6	102		60.71		± 14.33
**BMI–Males**	15.7	41		25.32		± 5.2
**BMI–Females**	14.8	41.3		24		± 5.25

The results were cross-tabulated. Pearson’s Chi-Square test was used to determine the association between the variables. Fisher exact test was used for variables having cells with an expected count less than 5. Statistical significance was set at p<0.05.

### Eating behaviors

The second section of the questionnaire consisted of items relating to the study participants’ eating behaviors, eating practices, and items assessing their awareness towards the health risks associated with obesity, the results of which are illustrated in Table 2.

**Table 2:** Crosstabulation of BMI with eating behaviors, eating practices, and awareness of the health risks associated with obesity.

Expected Answers	Under weight	Normal	Overweight	Obese	Total (Percent)	P-value
2.1 Eating behaviors						
**1. How often do you eat food/take snacks along with energy or carbonated drinks while watching Television?**						
**Everyday**	12 (21.8%)	74 (34.6%)	52 (61.2%)	34 (54.8%)	172 (41.3%)	p<0.001***
**3–4 times/week**	8 (14.5%)	33 (15.4%)	15 (17.6%)	17 (27.4%)	73 (17.5%)	
**1–2 times/week**	13 (23.6%)	15 (7.0%)	7 (8.2%)	6 (9.7%)	41 (9.9%)	
**Seldom/Rarely**	22 (40%)	92 (43.0%)	11 (12.9%)	5 (8.1%)	130 (31.3%)	
**2. How often do you eat food/take snacks along with energy or carbonated drinks while playing video games (or) games on your mobile phones?**						
**Everyday**	17 (30.9%)	37 (17.3%)	48 (56.5%)	41 (66.1%)	143 (34.4%)	p<0.001***
**3–4 times/week**	5 (9.1%)	26 (12.1%)	17 (20.0%)	5 (8.1%)	53 (12.7%)	
**1–2 times/week**	11 (20.0%)	51 (23.8%)	5 (5.9%)	7 (11.3%)	74 (17.8%)	
**Seldom/Rarely**	22 (40.0%)	100 (46.7%)	15 (17.6%)	9 (14.5%)	146 (35.1%)	
**3. How often do you take snacks separately from taking meals three times a day?**						
**Everyday**	21 (38.2%)	84 (39.3%)	44 (51.8%)	46 (74.2%)	195 (46.9%)	p<0.001***
**3–4 times/week**	22 (40.0%)	77 (36.0%)	31 (36.5%)	9 (14.5%)	139 (33.4%)	
**Seldom/Rarely**	12 (21.8%)	53 (24.8%)	10 (11.8%)	7 (11.3%)	82 (19.7%)	
**4. How often do you take energy drinks?**						
**Everyday**	12 (21.8%)	34 (15.9%)	22 (25.9%)	21 (33.9%)	89 (21.4%)	0.013 (p<0.05*)
**3–4 times/week**	11 (20.0%)	66 (30.8%)	24 (28.2%)	12 (19.4%)	113 (27.2%)	
**1–2 times/week**	24 (43.6%)	76 (35.5%)	34 (40.0%)	17 (27.4%)	151 (36.3%)	
**Seldom/Rarely**	8 (14.5%)	38 (17.8%)	5 (5.9%)	12 (19.4%)	63 (15.1%)	
**5. How often do you take carbonated or flavored drinks?**						
**Everyday**	18 (32.7%)	37 (17.3%)	44 (51.8%)	34 (54.8%)	133 (32.0%)	p<0.001***
**3–4 times/week**	7 (12.7%)	32 (15.0%)	27 (31.8%)	11 (17.7%)	77 (18.5%)	
**1–2 times/week**	19 (34.5%)	55 (25.7%)	6 (7.1%)	10 (16.1%)	90 (21.6%)	
**Seldom/Rarely**	11 (20.0%)	90 (42.1%)	8 (9.4%)	7 (11.3%)	116 (27.9%)	
**6. How often do you eat fruits and vegetables that are high on fiber?**						
**Everyday**	8 (14.5%)	22 (10.3%)	11 (12.9%)	10 (16.1%)	51 (12.3%)	0.332
**3–4 times/week**	6 (10.9%)	42 (19.6%)	11 (12.9%)	9 (14.5%)	68 (16.3%)	
**1–2 times/week**	32 (58.2%)	135 (63.1%)	55 (64.7%)	35 (56.5%)	257 (61.8%)	
**Seldom/Rarely**	9 (16.4%)	15 (7.0%)	8 (9.4%)	8 (12.9%)	40 (9.6%)	
**7. How often do you eat home-cooked food with your family?**						
**Everyday**	19 (34.5%)	59 (27.6%)	18 (21.2%)	14 (22.6%)	110 (26.4%)	0.244
**3–4 times/week**	13 (23.6%)	52 (24.3%)	28 (32.9%)	23 (37.1%)	116 (27.9%)	
**1–2 times/week**	14 (25.5%)	78 (36.4%)	24 (28.2%)	17 (27.4%)	133 (32.0%)	
**Seldom/Rarely**	9 (16.4%)	25 (11.7%)	15 (17.6%)	8 (12.9%)	57 (13.7%)	
**8. How often do you eat fast food?**						
**Everyday**	8 (14.5%)	34 (15.9%)	30 (35.3%)	13 (21.0%)	85 (20.4%)	p<0.001***
**3–4 times/week**	22 (40.0%)	60 (28.0%)	34 (40.0%)	27 (43.5%)	143 (34.4%)	
**1–2 times/week**	15 (27.3%)	31(14.5%)	14 (16.5%)	11 (17.7%)	71 (17.1%)	
**Seldom/Rarely**	10 (18.2%)	89 (41.6%)	7 (8.2%)	11 (17.7%)	117 (28.1%)	
**2.2 Eating practices**						
**9. How do you eat your food?**						
**On Dining Table**	31 (56.4%)	80 (37.4%)	61 (71.8%)	52 (83.9%)	224 (53.8%)	p<0.001***
**Islamic way of squatting on the ground**	24 (43.6%)	134 (62.6%)	24 (28.2%)	10 (16.1%)	192 (46.2%)	
**10. Do you take meals three times in a day?**						
**Yes**	47 (85.5%)	200 (93.5%)	73 (85.9%)	53 (85.5%)	373 (89.7%)	0.077
**No**	8 (14.5%)	14 (6.5%)	12 (14.1%)	9 (14.5%)	43 (10.3%)	
**11. Do you indulge in midnight snacking?**						
**Yes**	10 (18.2%)	48 (22.4%)	56 (65.9%)	36 (58.1%)	150 (36.1%)	p<0.001***
**No**	45 (81.8%)	166 (77.6%)	29 (34.1%)	26 (41.9%)	266 (63.9%)	
**12. Do you sleep immediately after having dinner?**						
**Yes**	22 (40.0%)	59 (27.6%)	30 (35.3%)	34 (54.8%)	145 (34.9%)	0.001 p<0.01**
**No**	33 (60.0%)	155 (72.4%)	55 (64.7%)	28 (45.2%)	271 (65.1%)	
**13. Do you walk for a while after having dinner?**						
**Yes**	16 (29.1%)	64 (29.9%)	19 (22.4%)	6 (9.7%)	105 (25.2%)	0.011 (p<0.05*)
**No**	39 (70.9%)	150 (70.1%)	66 (77.6%)	56 (90.3%)	311 (74.8%)	
**2.3 Physical activity**						
**14. How often do you exercise?**						
**Everyday**	13 (23.6%)	78 (36.4%)	11 (12.9%)	7 (11.3%)	109 (26.2%)	p<0.001***
**3–4 times/week**	6 (10.9%)	68 (31.8%)	22 (25.9%)	6 (9.7%)	102 (24.5%)	
**1–2 times/week**	15 (27.3%)	26 (12.1%)	18 (21.2%)	12 (19.4%)	71 (17.1%)	
**Seldom/Rarely**	21 (38.2%)	42 (19.6%)	34 (40.0%)	37 (59.7%)	134 (32.2%)	
**15. What kind of exercise do you do?**						
**Walking**	10 (18.2%)	29 (13.6%)	25 (29.4%)	20 (32.3%)	84 (20.2%)	p<0.001***
**Running**	17 (30.9%)	59 (27.6%)	8 (9.4%)	5 (8.1%)	89 (21.4%)	
**Swimming**	13 (23.6%)	64 (29.9%)	11 (12.9%)	7 (11.3%)	95 (22.8%)	
**Workout (Gym)**	6 (10.9%)	52 (24.3%)	8 (9.4%)	6 (9.7%)	72 (17.3%)	
**Do not like to Exercise**	9 (16.4%)	10 (4.7%)	33 (38.8%)	24 (38.7%)	76 (18.3%)	
**2.4 Awareness of the health risks associated with obesity**						
**16.1: Metabolic syndrome**						
**Yes**	10 (18.2%)	27 (12.6%)	13 (15.3%)	4 (6.5%)	54 (13.0%)	0.252
**No**	45 (81.8%)	187 (87.4%)	72 (84.7%)	58 (93.5%)	362 (87.0%)	
**16.2: Type—2 Diabetes**						
**Yes**	35 (63.6%)	147 (68.7%)	45 (52.9%)	36 (58.1%)	263 (63.2%)	0.062
**No**	20 (36.4%)	67 (31.3%)	40 (47.1%)	26 (41.9%)	153 (36.8%)	
**16.3: Hypertension**						
**Yes**	36 (65.5%)	167 (78.0%)	49 (57.6%)	47 (75.8%)	299 (71.9%)	0.003 p<0.01**
**No**	19 (34.5%)	47 (22.0%)	36 (42.4%)	15 (24.2%)	117 (28.1%)	
**16.4: Coronary Artery disease and Stroke**						
**Yes**	42 (76.4%)	149 (69.6%)	46 (54.1%)	42 (67.7%)	279 (67.1%)	0.026 (p<0.05*)
**No**	13 (23.6%)	65 (30.4%)	39 (45.9%)	20 (32.3%)	137 (32.9%)	
**16.5: Respiratory Disorders**						
**Yes**	15 (27.3%)	22 (10.3%)	11 (12.9%)	16 (25.8%)	64 (15.4%)	0.001 p<0.01**
**No**	40 (72.7%)	192 (89.7%)	74 (87.1%)	46 (74.2%)	352 (84.6%)	
**16.6: Reproductive Disorders**						
**Yes**	6 (10.9%)	6 (2.8%)	17 (20.0%)	11 (17.7%)	40 (9.6%)	p<0.001***
**No**	49 (89.1%)	208 (97.2%)	68 (80.0%)	51 (82.3%)	376 (90.4%)	
**16.7: Osteoarthritis**						
**Yes**	25 (45.5%)	46 (21.5%)	30 (35.3%)	26 (41.9%)	127 (30.5%)	p<0.001***
**No**	30 (54.5%)	168 (78.5%)	55 (64.7%)	36 (58.1%)	289 (69.5%)	
**16.8: Liver and Gallbladder diseases**						
**Yes**	9 (16.4%)	32 (15.0%)	21 (24.7%)	18 (29.0%)	80 (19.2%)	0.040 (p<0.05*)
**No**	46 (83.6%)	182 (85.0%)	64 (75.3%)	44 (71.0%)	336 (80.8%)	
**16.9: All of the above**						
**Yes**	6 (10.9%)	16 (7.5%)	13 (15.3%)	6 (9.7%)	41 (9.9%)	0.234
**No**	49 (89.1%)	198 (92.5%)	72 (84.7%)	56 (90.3%)	375 (90.1%)	
**16.10: None of the above**						
**Yes**	40 (72.7%)	149 (69.6%)	71 (83.5%)	43 (69.4%)	303 (72.8%)	0.093
**No**	15 (27.3%)	65 (30.4%)	14 (16.5%)	19 (30.6%)	113 (27.2%)	

*** denotes p<0.001

** denotes p<0.01

* denotes p<0.05

In the present study, it was observed that the overweight participants ate food/took snacks along with energy or carbonated drinks while watching television either daily (61.2%) or at least 3–4 times/week (17.6%), while the obese students did the same daily (54.8%) or at least 3–4 times/week (27.4%) *(p<0*.*001)*. Similarly, it was also observed that a very high percentage of the obese students (66.1%) ate food/took snacks along with energy or carbonated drinks while playing video games (or) games on their mobile phones daily *(p<0*.*001)*. It was also seen that participants who are obese (74.2%) and overweight (51.8%) took snacks three times a day, along with regular meals *(p<0*.*001)*. An alarming finding of this study was the daily consumption of energy drinks on their own among the obese (33.9%) and overweight (25.9%) students. The percentage of the students who consumed the same at least 3–4 times/week was 19.4% for obese and 28.2% for overweight students, respectively *(p = 0*.*013)*.

A similar bothersome finding of the current study is the daily consumption of carbonated or flavored drinks amongst the university students. More than half of the participants classified as obese (54.8%) and overweight (51.8%) consumed carbonated or flavored drinks daily*(p<0*.*001)*. For the consumption of fruits and vegetables that are rich in fiber, no statistically significant observation was shown *(p = 0*.*332)*. A similar non-significant finding was shown with regards to eating home-cooked food with family *(p = 0*.*244)*. One more unhealthy eating behavior was highlighted from the observation that the obese participants ate fast food daily (22.6%) or at least three to four times per week (43.5%) *(p<0*.*001)*.

### Eating practices

The students were asked as to how they took their food, i.e., on the dining table (or) in the Islamic way of squatting on the ground. It was observed that 83.9% of the obese and 71.8% of the overweight students ate their food on the dining table. While on the contrary, out of the 214 study participants who had normal weight, 62.6% ate their food in the Islamic way of squatting on the ground *(p<0*.*001)*. Taking meals three times a day did not yield any strong association between the observed groups *(p = 0*.*077)*. A common unhealthy eating practice among the overweight (65.9%) and obese (58.1%) study participants was midnight snacking *(p<0*.*001)*. It was also noticed that a very high proportion of obese students (54.8%) slept directly after dinner. Likewise, 35.3% of the students deemed as overweight also exhibited the same unhealthy practice *(p<0*.*01)*. Conversely, this practice was unseen among the students with normally distributed weight (72.4%). One more unhealthy practice seen across all weight categories was not walking after dinner. The highest percentages were seen amongst the obese (90.3%) and overweight students (77.6%). Surprisingly, this unhealthy practice was also seen among students with normal weight (70.1%) and those who were underweight (70.9%) *(p<0*.*05)*. 59.7% of obese, along with 40% of overweight participants seldom or rarely did any kind of exercise *(p<0*.*001)*. Also, when asked about the type of exercise, 38.7% of obese and 38.8% of overweight students did not like to exercise *(p<0*.*001)*.

### Awareness of health risks associated with obesity

A very high proportion of the study participants were ill-informed and unmindful that respiratory disorders (84.6%) *(p<0*.*01)*, reproductive disorders (90.4%) *(p<0*.*001)*, along with liver and gallbladder diseases (80.8%) *(p<0*.*05)* are some of the many health risks associated with obesity. The responses to the other risks are elucidated in Table 2.

“Table 3”, reflects the results of multiple logistic regression. It was observed that college students who live in rural areas were less likely to be obese than those who lived in urban areas (AOR: 0.20 C.I 95%; 0.06–0.63; p<0.01). Quite surprisingly, it was observed in our study sample that past smokers were 0.05 times less likely to be obese than the non-smokers. (AOR: 0.05 C.I 95%; 0.01–0.52; p<0.05). It was also noted that the students who ate food/took snacks along with energy or carbonated drinks daily while playing video games on mobile phones or computers were 7.11 more likely to be obese as compared to those who do this rarely (AOR: 7.11; C.I 95%: 2.40–21.07; p<0.001). It was also seen that students who consumed snacks everyday apart from meals were 11.52 times more likely to be obese in comparison to those who were seldom involved in this type of unhealthy eating behavior. (AOR: 11.52; C.I 95%: 3.04–43.63; p<0.001). The students consuming energy drinks everyday were 6.45 times more likely to be obese as compared to students who rarely consumed them. (AOR: 6.45; CI 95%: 1.58–26.43; p<0.05). Similarly, consumption of carbonated (or) flavored drinks daily made the students 8.85 times more likely to be obese in comparison with those who rarely indulged in this type of unhealthy eating behavior (AOR: 8.85; CI 95%: 2.53–30.90; p<0.01). One unique finding of this study showed that the students who ate their food on the dining table were 6.78 times more likely to be obese than those who ate food in the traditional Islamic way of squatting on the ground (AOR: 6.78; C.I 95%: 2.57–17.83; p<0.001). Additionally, the students who did not indulge in midnight snacking (AOR: 0.08; C.I 95%: 0.03–0.23; p<0.001) were less likely to be obese as compared to those who indulged in this kind of unhealthy eating practice. Likewise, with regards to the type of exercise frequented by the students, it was observed that those who worked out in the gym were less likely to be obese than those who did not like to exercise. (AOR: 0.09; C.I 95%: 0.02–0.40; p<0.01).

**Table 3:** Odds of overweight/obesity associated with selected eating behaviors and eating practices amongst sample of university students in Jazan, Saudi Arabia.

**S.No**	**Determinant**	**Adjusted Odds Ratio (AOR)**	**95% C.I**			**P-value**
			**Lower**	**Upper**		
1	Male	2.05	0.72	5.85		0.18
	Female					***Ref***
2	Age	0.80	0.65	1.00		0.05
3	Rural	0.20	0.06	0.63		0.006
	Urban					***Ref***
4	**Smoking History**					0.04
	Past Smoker	0.05	0.01	0.52		0.01
	Current Smoker	0.07	0.01	0.72		0.03
	Non Smoker					***Ref***
5	**Eating food/taking snacks along with energy or carbonated drinks while playing video games (or) games on your mobile phones**					p<0.001
	Everyday	7.11	2.40	21.07		p<0.001
	3–4 times/week	7.84	2.14	28.67		0.002
	1–2 times/week	0.60	0.16	2.34		0.47
	Seldom/Rarely					***Ref***
6	**Consumption of snacks apart from meals**					0.002
	Everyday	11.52	3.04	43.63		p<0.001
	3–4 times/week	6.65	1.67	26.54		0.007
	Seldom/Rarely					***Ref***
7	**Consumption of Energy drinks**					0.03
	Everyday	6.45	1.58	26.43		0.01
	3–4 times/week	2.27	0.55	9.32		0.25
	1–2 times/week	6.08	1.48	24.94		0.01
	Seldom/Rarely					***Ref***
8	**Consumption of carbonated (or) flavored drinks**					p<0.001
	Everyday	8.85	2.53	30.90		0.001
	3–4 times/week	8.01	2.02	31.75		0.003
	1–2 times/week	1.09	0.25	4.80		0.91
	Seldom/Rarely					***Ref***
9	**Consumption of high fiber fruits and vegetables**					0.70
	Everyday	0.97	0.14	6.82		0.98
	3–4 times/week	1.83	0.29	11.46		0.52
	1–2 times/week	0.85	0.15	4.71		0.86
	Seldom/Rarely					***Ref***
10	**Consumption of home cooked food**					0.07
	Everyday	0.48	0.10	2.26		0.36
	3–4 times/week	2.09	0.46	9.54		0.34
	1–2 times/week	1.34	0.27	6.55		0.72
	Seldom/Rarely					***Ref***
11	**Consumption of fast food**					0.30
	Everyday	3.49	0.89	13.74		0.07
	3–4 times/week	1.91	0.55	6.65		0.31
	1–2 times/week	1.23	0.27	5.56		0.79
	Seldom/Rarely					***Ref***
12	**Way of eating food**					
	Dining Table	6.78	2.57	17.83		p<0.001
	Islamic way of Squatting on the ground					***Ref***
13	**Taking meals three times a day**					
	No	0.47	0.12	1.87		0.29
	Yes					***Ref***
14	**Midnight Snacking**					
	No	0.08	0.03	0.23		p<0.001
	Yes					***Ref***
15	**Sleeping immediately after dinner**					
	No	0.66	0.26	1.67	0.38	
	Yes				***Ref***	
16	**Walking after dinner**					
	No	3.23	1.00	10.39		0.05
	Yes					***Ref***
17	**Frequency of Exercise**					0.22
	Everyday	0.29	0.08	1.08		0.06
	3–4 times/week	0.31	0.08	1.16		0.08
	1–2 times/week	0.57	0.18	1.82		0.34
	Seldom/Rarely					***Ref***
18	**Type of Exercise**					p<0.001
	Walking	0.29	0.07	1.08		0.07
	Running	0.02	0.003	0.10		p<0.001
	Swimming	0.03	0.007	0.16		p<0.001
	Workout (Gym)	0.09	0.02	0.40		0.001
	**Do not like to Exercise**					***Ref***

C. I—Confidence Interval

The variables that were included in the multiple logistic regression model were gender, age, location, smoking history, as well as consumption of food/snacks along with energy or carbonated drinks while playing video games, consumption of snacks apart from meals, consumption of energy drinks, carbonated (or) flavored drinks, consumption of fruits and vegetables high on fiber, home cooked food, fast food, way of eating, taking meals three times a day, midnight snacking, sleeping immediately after dinner, walking for a while after dinner, frequency of exercise and type of exercise.

## Discussion

It was observed in the current study that prevalence of obesity (14.9%) and overweight (20.4%) among the 416 study sample were relatively consistent with Al Reithaa et al. who also found a fairly similar percentage of obesity (15.7%) and overweight (21.8%) in a 357 study sample [3]. The distribution of the students among the normal weight was fairly alike across the genders, whereas underweight was more common among the female students. Yahia et al., also made a similar observation of an increased percentage of underweight amongst female university students [18]. This could be explained by the fact that the westernization of Saudi society is making the younger generation, especially the females, more conscious about their weight and body image. With regards to the different eating behaviors, it was seen that the consumption of food/snacks along with energy or carbonated drinks while watching television as well as while playing videogames (or) games on mobile phones was comparatively higher amongst the obese and the overweight participants. A.O Musaiger et al. J Vioque et al. and Liang, T etal. were also able to find a similar positive association between watching television and obesity [19–21]. Al-Hazzaa HM et al. attributed watching television along with playing games on the computer and video games as key contributors to physical inactivity [15]. MB Neutzling et al. also regarded the time spent while watching television, playing video games or using the computer as indirect indicators of sedentary lifestyle along with having an association with overweight as well as obesity [22]. The more the time spent in inactivity and immobility while watching television, playing videogames (or) games on mobile phones, and eating/ drinking unhealthy food while doing so, the less likely it is for the person to indulge in any kind of physical activity. We were also able to establish an association between consumption of energy drinks while watching television, playing video games (or) games on mobile phones and obesity. Snacking everyday separately from taking meals three times a day was also seen among the obese and overweight students, thus drawing a similarity with Al-Rethaiaa et al. [3]. The consumption of energy drinks has become a common trend amongst college going students [23]. We also made a similar observation in our study wherein we found that more than a quarter of the obese and overweight students consumed energy drinks daily. The probable reason for the high intake of energy drinks among our students could be owing to the stringent and compact schedule of the semesters, with intense and stressful situations arising because of frequent exams, pop quizzes, assignments and project works. In a study done by Kabir A et al. the investigators noted that exam stress has a marked influence on the eating habits of the students [24]. A similar finding was also reported by Deliens et al [25]. wherein the study participants reported their eating behaviors to differ during the academic year and the exams. We were also able to establish a significant association between consuming carbonated or flavored drinks and obesity. David S Ludwig et al. concluded that the intake of drinks sweetened by sugar could possibly lead to obesity [26]. Similar to R. Tumin, S. E. Anderson, and Bin Zaal A. A. et al. we were also unable to find any significant association between obesity and having meals with the family [27, 28]. A similar finding between obesity and staying with family was also seen by Tanton J et al. amongst British university students, it was reported that 82% of the students lived off-campus, thus highlighting the fact that staying on campus may not be the only contributing factor for obesity [29]. El Ansari et al. who conducted a study exploring the correlation between consumption of food with their living arrangements amongst university students of four European countries, also reported a similar finding saying that snacks and fast food consumption were independent of living arrangements [30]. According to previously conducted studies across some US universities, it was observed that students did not consume fruits as well as vegetables in their recommended amounts, whilst also observing that food containing high levels of fats were being consumed at an increased level [31–33]. We also made a similar observation that more than half of the students deemed overweight and obese consumed fruits and vegetables rich in fiber just once or twice per week. In the present study, a statistically significant association between the consumption of fast food and obesity was also seen. Butler et al. also made a similar observation in terms of less consumption of vegetables and increased consumption of fatty foods and alcohol during the initial years of university life [32].

It is imperative to make concerted efforts aimed at educating the students by making use of seminars, presentations, and symposiums highlighting the deleterious risks posed by the various unhealthy eating behaviors and their significant association in increasing the incidence of obesity. Tanton J et al. also made a similar inference, while studying the British university students. He found a discrete cluster pattern with regards to eating behaviours. Nearly 31.6% of the sample fell under the clusters of risky eating behavior and mixed eating behaviors. These are the 2 clusters that which show the eating behaviors deemed the riskiest [29]. A high prevalence of eating behaviors deemed unhealthy, urgently necessitates interventions among the British university students to encourage healthy eating behaviors [29].

To the best of authors’ knowledge, this is the first study to investigate and establish an association between obesity and the way in which food is eaten, i.e., the modern way in which food is eaten on the dining table or the traditional Islamic way in which it is eaten while squatting on the ground, which is still prevalent in Arab countries. One of the unique findings of our study was our observation wherein we found a very high proportion of the obese and overweight students eating their food on the dining table. While on the contrary, a similarly high proportion of the students with normal weight ate their food by squatting on the ground, thus establishing a strong association between obesity and eating the food on the dining table. The probable reason for this could be that, it is a common eating practice among the Muslims to squat down with either one (or) both of their legs pressing the stomach. Eating in such a position coupled with the pressure caused by the legs pressing against the stomach may cause a false sense of fullness and thus prevents overeating. Mid-night snacking was seen as a common unhealthy practice among many of the male study participants. Bin Zaal A. A. et al. also reported a similar finding [28]. One more unique finding of our study was establishing an association between obesity and an unhealthy practice like sleeping directly after dinner. Walking for some time after dinner was an uncommon practice.

Additionally, it was also observed that nearly most of the students were oblivious to the fact that metabolic syndrome, reproductive disorders, respiratory disorders along with liver and gallbladder diseases are some of the many health risks associated with obesity. Sedentary lifestyle, coupled with unhealthy eating practices, is a dangerous combination. It is crucial to encourage the students to alter their unhealthful eating practices along with inactive lifestyles and also to persuade them to increase their physical activity to reduce any possible health risks posed by obesity and also gain further information on these obesity-associated risk factors.

The strengths of the study include the recruitment of both the genders, while many previously conducted studies had a gender bias wherein they included only the male students. As Jazan University is the only university in the whole of the province, students from different socio-demographic characteristics were included and studied. Even though there are a few studies which have previously assessed the eating behaviors among the university students, eating practices remained relatively untouched and our assessment of the eating practices in our study sample, along with our assessment of the student’s awareness of the health risks associated with obesity should be considered as our uniqueness.

### Limitations

Some of the limitations of the study include the inability to establish a causal relationship owing to its cross-sectional study design. The study was conducted in one university and only amongst its health science colleges. The sample size was only 416 students. Moreover, the daily caloric consumption, duration, intensity of physical activity, quality and duration of sleep were not included in the questionnaire. Furthermore, with a convenience sampling technique we cannot claim that our sample is a representative of a larger population. Moreover, all the study participants were of the same race (Arabs). Due to the conservative nature of the study participants from Jazan, most did not consent to record their hip and waist measurements, hence we were unable to calculate the hip and waist ratio. Moreover, due to shyness and stigma attached to being categorized as obese, many female students answered the questionnaire but did not complete the study as they did not consent to record their weight. Some of the participants withdrew their consent after completing the study as they were embarrassed to see their readings being way above the normal limits. Some of the responses could also be subject to recall bias.

## Conclusions

The prevalence of obesity and overweight were found to be reasonably high in our study sample and were affected by several factors related to eating behaviors and practices. Moreover, it was also observed that nearly most of the students were oblivious to the fact that metabolic syndrome, reproductive disorders, respiratory disorders along with liver and gallbladder diseases are some of the many health risks associated with obesity. Increasing trends of obesity amongst the college students warrant a pressing and urgent need to provide health education to the students towards the health risks associated with obesity. Moreover, the findings also suggest the need for further rigorous and frequent health education interventions concentrating on promoting healthy eating behaviors, eating practices along with a special emphasis on the importance of adopting an active, healthy lifestyle. Counseling the students seriously, honestly, and urgently on the imperative need to change their eating behaviors, practices, and sedentary lifestyles is the need of the hour.

## Supporting information

S1 Questionnaire English (DOCX)

S2 Questionaire Arabic (DOCX)

S1 Informed consent (PDF)
