# Supplementary material for: The association of dietary behaviors and practices with overweight and obesity parameters among Saudi university students
Source: PLoS One. 2020 Sep 10;15(9):e0238458. doi: 10.1371/journal.pone.0238458 (PMC7482918; doi:10.1371/journal.pone.0238458)
Supplement: S1 Questionnaire English — (DOCX) [file pone.0238458.s001.docx]

**General Instructions for use:**

1. The Questionnaire contains **TWO** sections, kindly answer all the questions EARNESTLY.

2. Kindly tick the most appropriate answer. Use legible ink. & do not over-write.

**SECTION - 1: DEMOGRAPHICS**

| **S.No** | **CATEGORY** | **OPTIONS** | **TICK HERE** |
| --- | --- | --- | --- |
|  | | | |
| **D1** | **Gender:** | **1. Male** | **🞏** |
|  |  | **2. Female** | **🞏** |
| **D2** | **Age Group in years:** | **1: 18 - 20** | **🞏** |
|  |  | **2: 21 - 23** | **🞏** |
|  |  | **3: 24 - 26** | **🞏** |
| **D3** | **Age in years:** | **………………………………..** | |
| **D4** | **Nationality:** | **1. Saudi** | **🞏** |
|  |  | **2. Non - Saudi** | **🞏** |
| **D5** | **College:** | **1: Pharmacy College** | **🞏** |
|  |  | **2. Medical College** | **🞏** |
|  |  | **3. Dental College** | **🞏** |
|  |  | **4. Nursing College** | **🞏** |
|  |  | **5. Applied Health College** | **🞏** |
|  |  | **6. Public Health** | **🞏** |
| **D6** | **Level:** | **1: 3^rd^ Year** | **🞏** |
|  |  | **2. 4^th^ Year** | **🞏** |
|  |  | **3: 5^th^ Year** | **🞏** |
|  |  | **4: 6^th^ Year** | **🞏** |
| **D7** | **Location** | **0: Urban** | **🞏** |
|  |  | **1: Rural** | **🞏** |
| **D8** | **Smoking History:** | **1: Past Smoker** | **🞏** |
|  |  | **2: Current Smoker** | **🞏** |
|  |  | **3: Non - Smoker** | **🞏** |
|  | | | |

| **ANTHROPOMETRIC MEASUREMENTS** | | | | |
| --- | --- | --- | --- | --- |
| **D9** | **1. Height: …………… (cms) 2. Weight: …………... (Kgs) 3. BMI: …………….** | | | |
| **D10** | **BMI CATEGORY** | **1: Under weight (< 18.5)** | **🞏** |  |
|  |  | **2. Normal (18.5 - 24.9)** | **🞏** |  |
|  |  | **3: Overweight (25 - 29.9)** | **🞏** |  |
|  |  | **4: Obese (> 30)** | **🞏** |  |

| **S.No** | | **QUESTIONS** | | **EXPECTED ANSWERS** | **TICK HERE** | |
| --- | --- | --- | --- | --- | --- | --- |
| **SECTION – 2.1: EATING BEHAVIOURS** | | | | |  | |
| **1.** | | **How often do you eat food/take snacks along with energy or carbonated drinks while watching Television? n** | | 1. Every day. | **🞏** | |
|  |  |  |  | 2. 3-4 times/week. | **🞏** | |
|  |  |  |  | 3. 1-2 times/week. | **🞏** | |
|  |  |  |  | 4. Seldom/Rarely. | **🞏** | |
| **2.** | | **How often do you eat food/take snacks along with energy or carbonated drinks while playing video games (or) games on your mobile phones?** | | 1. Every day. | **🞏** | |
|  |  |  |  | 2. 3-4 times/week. | **🞏** | |
|  |  |  |  | 3. 1-2 times/week. | **🞏** | |
|  |  |  |  | 4. Seldom/Rarely. | **🞏** | |
| **3.** | | **How often do you take snacks separately from taking meals thrice a day?** | | 1. Every day. | **🞏** | |
|  |  |  |  | 2. 3-4 times/week. | **🞏** | |
|  |  |  |  | 3. 1-2 times/week. | **🞏** | |
|  |  |  |  | 4. Seldom/Rarely. | **🞏** | |
| **4.** | | **How often do you take energy drinks?** | | 1. Every day. | **🞏** | |
|  |  |  |  | 2. 3-4 times/week. | **🞏** | |
|  |  |  |  | 3. 1-2 times/week. | **🞏** | |
|  |  |  |  | 4. Seldom/Rarely. | **🞏** | |
| **5.** | | **How often do you take carbonated or flavored drinks?** | | 1. Every day. | **🞏** | |
|  |  |  |  | 2. 3-4 times/week. | **🞏** | |
|  |  |  |  | 3. 1-2 times/week. | **🞏** | |
|  |  |  |  | 4. Seldom/Rarely. | **🞏** | |
| **6.** | | **How often do you eat fruits and vegetables that are high on fiber?** | | 1. Every day. | **🞏** | |
|  |  |  |  | 2. 3-4 times/week. | **🞏** | |
|  |  |  |  | 3. 1-2 times/week. | **🞏** | |
|  |  |  |  | 4. Seldom/Rarely. | **🞏** | |
| **7.** | | **How often do you eat home-cooked food with your family?** | | 1. Every day. | **🞏** | |
|  |  |  |  | 2. 3-4 times/week. | **🞏** | |
|  |  |  |  | 3. 1-2 times/week. | **🞏** | |
|  |  |  |  | 4. Seldom/Rarely. | **🞏** | |
| 8. | | **How often do you eat fast food?** | | 1. Every day. | **🞏** | |
|  |  |  |  | 2. 3-4 times/week. | **🞏** | |
|  |  |  |  | 3. 1-2 times/week. | **🞏** | |
|  |  |  |  | 4. Seldom/Rarely. | **🞏** | |
| **SECTION – 2.2: EATING PRACTICES** | | | | | | |
| 9. | | **How do you eat your food?** | | 0. On a dining table. | **🞏** | |
|  |  |  |  | 1. Islamic way of Squatting down. | **🞏** | |
| 10. | | **Do you take meals three times in a day?** | | 0. No. | **🞏** | |
|  |  |  |  | 1. Yes. | **🞏** | |
| 11. | | **Do you indulge in midnight snacking?** | | 0. No. | **🞏** | |
|  |  |  |  | 1. Yes. | **🞏** | |
| 12. | | **Do you sleep immediately after having dinner?** | | 0. No. | **🞏** | |
|  |  |  |  | 1. Yes. | **🞏** | |
| 13. | | **Do you walk for a while after dinner?** | | 0. No. | **🞏** | |
|  |  |  |  | 1. Yes. | **🞏** | |
| **SECTION – 2.3: PHYSICAL ACTIVITY** | | | | | | |
| 14. | **How often do you exercise?** | | | 1. Every day | | **🞏** |
|  |  |  |  | 2. 3-4 times/week. | | **🞏** |
|  |  |  |  | 3. 1-2 times/week. | | **🞏** |
|  |  |  |  | 4. Seldom/Rarely. | | **🞏** |
| 15. | **What kind of exercise do you do?** | | | 1. Walking | | **🞏** |
|  |  |  |  | 2. Running / Cycling | | **🞏** |
|  |  |  |  | 3. Swimming | | **🞏** |
|  |  |  |  | 4. Workout in the Gym | | **🞏** |
|  |  |  |  | 5. Do not like to Exercise | | **🞏** |
| **SECTION – 2.4: AWARENESS OF RISKS ASSOCIATED WITH OBESITY** | | | | | | |
| 16. | **Which of the following do you think are the risks associated with Obesity?** | | **1. Metabolic syndrome.** | 0. No. | **🞏** | |
|  |  |  |  | 1. Yes. | **🞏** | |
|  |  |  | **2. Type – 2 Diabetes.** | 0. No. | **🞏** | |
|  |  |  |  | 1. Yes. | **🞏** | |
|  |  |  | **3. Hypertension.** | 0. No. | **🞏** | |
|  |  |  |  | 1. Yes. | **🞏** | |
|  |  |  | **4. Coronary artery disease (CAD) & Stroke.** | 0. No. | **🞏** | |
|  |  |  |  | 1. Yes. | **🞏** | |
|  |  |  | **5. Respiratory disorders.** | 0. No. | **🞏** | |
|  |  |  |  | 1. Yes. | **🞏** | |
|  |  |  | **6. Reproductive disorders.** | 0. No. | **🞏** | |
|  |  |  |  | 1. Yes. | **🞏** | |
|  |  |  | **7. Osteoarthritis (OA).** | 0. No. | **🞏** | |
|  |  |  |  | 1. Yes. | **🞏** | |
|  |  |  | **8. Liver & Gall bladder disease.** | 0. No. | **🞏** | |
|  |  |  |  | 1. Yes. | **🞏** | |
|  |  |  | **9. All of the above.** | 0. No. | **🞏** | |
|  |  |  |  | 1. Yes. | **🞏** | |
|  |  |  | **10. None of the above.** | 0. No. | **🞏** | |
|  |  |  |  | 1. Yes. | **🞏** | |

**THANK YOU VERY MUCH INDEED FOR YOUR TIME & PATIENCE IN COMPLETING THE SURVEY.**
