# Supplementary material for: The association of dietary behaviors and practices with overweight and obesity parameters among Saudi university students
Source: PLoS One. 2020 Sep 10;15(9):e0238458. doi: 10.1371/journal.pone.0238458 (PMC7482918; doi:10.1371/journal.pone.0238458)
Supplement: S2 Questionaire Arabic — (DOCX) [file pone.0238458.s002.docx]

**إستبيــان دراســة**

**تعليمات عامة للاستخدام:**

1. يحتويالاستبيان على جزأين، ويرجى التكرم بالإجابة على كل الاسئلة بجدية.
2. يرجى وضع علامة صح **(√)** على الإجابة الأكثر ملاءمة، مع استخدام الحبر الجاف وعدم الكشط.

| **الجزء الأول - 1: التركيبــــة السـكانيــة** | | | | | |  |  |  |  |
| --- | --- | --- | --- | --- | --- | --- | --- | --- | --- |
| **م** | **الفئـــة** | | **الخيـــارات** | | **ضع علامة (√)** |  |  |  |  |
| 1 | النوع: | | 1- ذكر | |  |  |  |  |  |
|  |  |  | 2- أنثى | |  |  |  |  |  |
| 2 | المجموعة العمرية (بالسنوات): | | 1: 18 - 20 | |  |  |  |  |  |
|  |  |  | 2: 21 - 23 | |  |  |  |  |  |
|  |  |  | 3: 24 - 26 | |  |  |  |  |  |
| 3 | العمر بالسنوات: | | ................................................ | | |  |  |  |  |
| 4 | الجنسية: | | 1. سعودي | |  |  |  |  |  |
|  |  |  | 2. غير سعودي | |  |  |  |  |  |
| 5 | الكليـــة: | | 1. كليـة الصيدلة | |  |  |  |  |  |
|  |  |  | 2. كليـة الطـــب | |  |  |  |  |  |
|  |  |  | 3. كليـة طـب الأسنان | |  |  |  |  |  |
|  |  |  | 4. كليـة التمريــض | |  |  |  |  |  |
|  |  |  | 5. كليـة العلوم الطبية التطبيقية | |  |  |  |  |  |
|  |  |  | 6. كليـة الصحة العــامة | |  |  |  |  |  |
| 6 | المستوى: | | 1. السـنة الثالثة | |  |  |  |  |  |
|  |  |  | 2. السـنة الرابعة | |  |  |  |  |  |
|  |  |  | 3. السـنة الخامسة | |  |  |  |  |  |
|  |  |  | 4. السـنة السادسة | |  |  |  |  |  |
| 7 | الموقـــع: | | 0- في مدينة | |  |  |  |  |  |
|  |  |  | 1- ريفـــي | |  |  |  |  |  |
| 8 | تاريخ التدخيــن: | | 1: مدخــن سابق | |  |  |  |  |  |
|  |  |  | 2: مدخــن حالي | |  |  |  |  |  |
|  |  |  | 3: غير مدخــن | |  |  |  |  |  |
| **قياســـــات الجســم البشـــري** | | | | | | | | | |
| 9 | 1. الطول: ............ (سم) 2. الوزن: ............. (كجم) 3. مؤشر كتلة الجسم .................... | | | | | | | | |
| 10 | فئــة مؤشـر كتلـة الجسـم: | | | 1: وزن أقل (‹ 18.5) | |  | | |  |
|  |  |  |  | 2: وزن طبيعي (18.5–14.9) | |  | | |  |
|  |  |  |  | 3 وزن زائد (25 – 29.9) | |  | | |  |
|  |  |  |  | 4: بديــــن (› 30) | |  | | |  |

| **م** | **الأســـــئلة** | **الإجـابــات المتوقعـــــة** | **ضـع (√)** |
| --- | --- | --- | --- |
| **الجزء الثاني-1: عــــادات الأكــــــل** | | |  |
| 1 | كم مرة تتناول الطعام/وجبات خفيفة مع مشروبات الطاقة أو المشروبات الغازية أثناء مشاهدة التلفاز؟ | 1. كــل يـــوم |  |
|  |  | 1. 3–4 مرات/الاسبوع |  |
|  |  | 1. 1–2 مرات/الاسبوع |  |
|  |  | 1. قليــلاً ما / نــادراً |  |
| 2 | كم مرة تتناول الطعام/وجبات خفيفة مع مشروبات الطاقة أو المشروبات الغازية أثناء ممارسة ألعاب الفيديو (أو) الألعاب في هاتفك الجوال؟ | 1. كــل يـــوم |  |
|  |  | 1. 3–4 مرات/الاسبوع |  |
|  |  | 1. 1–2 مرات/الاسبوع |  |
|  |  | 1. قليــلاً ما / نــادراً |  |
| 3 | كم مرة تتناول الوجبات الخفيفة منفصلة عن تناول الوجبات الثلاثة اليومية؟ | 1. كــل يـــوم |  |
|  |  | 1. 3–4 مرات/الاسبوع |  |
|  |  | 1. 1–2 مرات/الاسبوع |  |
|  |  | 1. قليــلاً ما / نــادراً |  |
| 4 | كم مرة تتناول مشروبات الطاقة؟ | 1. كــل يـــوم |  |
|  |  | 1. 3–4 مرات/الاسبوع |  |
|  |  | 1. 1–2 مرات/الاسبوع |  |
|  |  | 1. قليــلاً ما / نــادراً |  |
| 5 | كم مرة تتناول المشروبات الغازية أو المضاف إليها نكهات؟ | 1. كــل يـــوم |  |
|  |  | 1. 3–4 مرات/الاسبوع |  |
|  |  | 1. 1–2 مرات/الاسبوع |  |
|  |  | 1. قليــلاً ما / نــادراً |  |
| 6 | كم مرة تتناول الطعام المُعد في البيت مع عائلتك؟ | 1. كــل يـــوم |  |
|  |  | 1. 3–4 مرات/الاسبوع |  |
|  |  | 1. 1–2 مرات/الاسبوع |  |
|  |  | 1. قليــلاً ما / نــادراً |  |
| 7 | كم مرة تتناول الوجبـات السريعـة؟ | 1. كــل يـــوم |  |
|  |  | 1. 3–4 مرات/الاسبوع |  |
|  |  | 1. 1–2 مرات/الاسبوع |  |
|  |  | 1. قليــلاً ما / نــادراً |  |
| **الجزء الثاني-2: ممارســــات الأكــــــل** | | | |
| 8 | كيف تتناول طعامك؟ | 0. من على طاولة طعام |  |
|  |  | 1. جالساً القرفصاء على الأرض بالطريقة الاسلامية |  |
| 9 | هل تتناول الوجبات ثلاث مرات في اليوم؟ | 0. لا |  |
|  |  | 1. نعم |  |

| 10 | هل تقوم بتناول وجبات في منتصف الليل؟ | | | 0. لا |  |
| --- | --- | --- | --- | --- | --- |
|  |  |  |  | 1. نعم |  |
| 11 | هل تنام بعد تناول العشاء مباشرة؟ | | | 0. لا |  |
|  |  |  |  | 1. نعم |  |
| 12 | هل تتمشى قليلاً بعد تناول العشاء؟ | | | 0. لا |  |
|  |  |  |  | 1. نعم |  |
| **الجزء الثاني-3: النشــــاط الجسـمـــاني** | | | | | |
| 13 | كم مرة تقوم بأداء التمارين؟ | | | 4. كــل يـــوم |  |
|  |  |  |  | 3. 3–4 مرات/الاسبوع |  |
|  |  |  |  | 2. 1–2 مرات/الاسبوع |  |
|  |  |  |  | 1. قليــلاً ما / نــادراً |  |
| 14 | ما نوع التمارين التي تمارسها؟ | | | 1. المشــي |  |
|  |  |  |  | 2. الجري/ركوب الدراجة |  |
|  |  |  |  | 3. السباحــة |  |
|  |  |  |  | 4. التدرُب في صالة رياضية |  |
|  |  |  |  | 5. لا أحب التمــاريــن |  |
| **الجزء الثاني-4: الوعــي بالمخـاطر المرتبطـــة بالـسـمـنــة** | | | | | |
| 15 | أياً من المخـاطر التاليــة تعتقــد أنـها مرتبطـــة بالـسـمـنــة؟ | 1. متلازمة الأيض | | 0. لا |  |
|  |  |  |  | 1. نعم |  |
|  |  | 2. داء السكر من النوع الثاني | | 0. لا |  |
|  |  |  |  | 1. نعم |  |
|  |  | 3.ارتفاع الضغط | | 0. لا |  |
|  |  |  |  | 1. نعم |  |
|  |  | 4. مرض القلب التاجي والسكتة الدماغية | | 0. لا |  |
|  |  |  |  | 1. نعم |  |
|  |  | 5.اضطرابات الجهاز التنفسي | | 0. لا |  |
|  |  |  |  | 1. نعم |  |
|  |  | 6.الاضطرابات التناسلية | | 0. لا |  |
|  |  |  |  | 1. نعم |  |
|  |  | 7.التهاب العظام والعمود الفقري | | 0. لا |  |
|  |  |  |  | 1. نعم |  |
|  |  | 8. أمراض الكبد والمرارة | | 0. لا |  |
|  |  |  |  | 1. نعم |  |
|  |  | | 9. كل ما سبق ذكره | 0. لا |  |
|  |  |  |  | 1. نعم |  |
|  |  |  | 10. لا شيء مما سبق ذكره | 0. لا |  |
|  |  |  |  | 1. نعم |  |

***لكم جزيل الشكر على وقتكم الثمين وصبركم لإكمال هذا الاستبيان.***
