# Supplementary material for: The association of dietary behaviors and practices with overweight and obesity parameters among Saudi university students
Source: PLoS One. 2020 Sep 10;15(9):e0238458. doi: 10.1371/journal.pone.0238458 (PMC7482918; doi:10.1371/journal.pone.0238458)
Supplement: S1 Informed consent — (PDF) [file pone.0238458.s003.pdf]

استمارة طلب موافقة مسبقة

الهدف:
إن الهدف من استمارة الموافقة المسبقة هو اعلام المشاركين في الدراسة بهدف البحث وكيف أن بإمكانهم المشاركة في خلقِ وعيٍ عام نحو مواضيع محددة.

الفوائد: إن البرامج التعليمية يمكن أن تصُمم وتعُد لخِلقِ وعيٍ عام تجاه مختلف المواضيع اذا كان هناك معدل انخفاض لكلٍ من المعرفة و السلوك والممارسة والإعتقاد، وهذا ما سَنسُلطُ الضوء عليه في هذا البحث.

المخاطر: كون هذه الدراسة دراسة استقصائية مقطعية (تهدف للملاحظة) ليس هناك اي مخاطر او ماشابه على المشاركين بها.

الخصوصية/السرية: لن يكون لدينا أي وصول للبيانات الشخصيةِ: لذلك لن تكون هناك أي متاعب متعلقة بسرية البيانات. إن الغرض من المعلومات المطلوبة هو وبشكلٍ صارم لأبحاث الطلاب وللغرض العلمي فقط.

عنوان الدراسة: إرتباط السلوكيات والممارسات الغذائية بمعايير زيادة الوزن والسمنة بين طلاب الجامعات السعودية

الهدف من الدراسة:
عَمِدنا في هذه الدراسة إلى تحديدِ مستوى الوعي والسلوك والممارسة بين طلاب الجامعات السعودية. و عند رصدِ أي قصورٍ تتعلق بالمعرفة والسلوك والممارسة بإنتهاء هذه الدراسة، فإننا سَنعمَدُ إلى طرحِ أساليب فعالة للتغلب على هذه القصور من خلال مختلف البرامج التعليمية العامة وبالتالي نحقق الغرض المراد من هذا البحث، ألا وهو نشر الوعي تجاه مختلف المواضيع.

مشاركة طوعية (اختياريه) :
إن المشاركة في هذه الدراسة هو أمرٌ اختياريٌ بالكامل. اذا قررت المشاركة في هذه الدراسة فيجب أن يكون هذا نابعٌ من إرادتك الخاصة. أنت لست تحت أي ظروف تجبرك على المشاركة بهذه الدراسة. فضلاً كن على علم أنه اذا قررت عدم المشاركة فهذا أمرٌ مقبول. كن على علمٍ أيضا انه اذا قررت بأي لحظة عدم إكمال الإستبيان فلك الحرية بذلك. اذا شعرت بعدم الإرتياح مع أي سؤال فيمكنك الإمتناع عن الإجابة عليه.

بالتوقيع على استمارة الموافقة هذه ، فأنا أقر أنني قد قرأت وفهمت المعلومات المذكورة سابقاً (فوائد ومخاطر الدراسة) وأنني بإرادتي الخاصة أعطي موافقتي على المشاركة في الدراسة المذكورة، ولم أجبر بأي حالٍ من الأحوال على التوقيع على استمارة الموافقة هذهِ.

الإسم : .... التوقيع : ....

التاريخ : ....

وحدة أبحاث الممارسة الصيدلية
كلية الصيدلة
